# How I do it: Endoscopic microvascular decompression with vertebral artery transposition for trigeminal neuralgia caused by vertebrobasilar dolichoectasia

**DOI:** 10.1007/s00701-026-06940-1

**Published:** 2026-06-03

**Authors:** Viktoriia Kuts-Karpenko, Fuminari Komatsu, Yoko Kato

**Affiliations:** 1 Department of Neurosurgery, Municipal Non-Profit Enterprise “Lviv Territorial Medical Union Multidisciplinary Clinical Hospital of Emergency and Intensive Care” (First Lviv Territorial Medical Union), 9 I. Mykolaychuka St., Lviv, Ukraine; 2 Department of Neurosurgery, Fujita Health University Bantane Hospital, 3-6-10 Otobashi, Nakagawa-Ku, Nagoya, Aichi 454-8509 Japan

**Keywords:** Endoscopic keyhole surgery, Root entry zone, Vertebral artery, Vertebrobasilar dolichoectasia, Transposition

## Abstract

**Background:**

Vertebrobasilar dolichoectasia (VBDE) is an uncommon cause of trigeminal neuralgia (TN), in which an ectatic vertebral artery (VA) compresses the root entry zone (REZ), while perforators may limit safe transposition.

**Methods:**

Fully endoscopic microvascular decompression with VA transposition was performed. Preservation of all arterial branches was achieved and confirmed endoscopically.

**Conclusion:**

In VBDE-related TN, VA transposition may be the preferred decompressive strategy because of the characteristics of the ectatic vessel. Endoscopy offers a wide-angle view of the REZ, facilitating safe mobilization under full visualization of branch vessels.

**Supplementary Information:**

The online version contains supplementary material available at 10.1007/s00701-026-06940-1.

## Relevant surgical anatomy

VA-related trigeminal neuralgia (TN) is an uncommon variant, reported in approximately 2–7.7% of patients with classic TN [1]. From a hemodynamic perspective, in VBDE the left VA has a larger caliber and is exposed to greater pulse pressure, contributing to elongation and tortuosity. In left-sided VBDE the ectatic VA frequently produces more than simple contact with the trigeminal nerve (Grade I) and may cause deviation (Grade II) or deep indentation (Grade III) with nerve thinning or atrophy [5]. In such cases, the caliber and rigidity of the offending vessel and the severity of compression necessitate complete separation of the artery from the REZ, rather than limited interposition.

Safe decompression requires reliable visualization of the trigeminal nerve (CN V) REZ, the ectatic VA loop and its short perforating branches, and adjacent CPA structures defining the working corridor. VBDE with an ectatic VA can also alter the course and origin of the anterior spinal artery (ASA); identifying and confirming the absence of the ASA within the planned transposition corridor is important to avoid inadvertent injury. Endoscopy facilitates inspection of the vessel circumference and the REZ corridor to confirm branch safety during mobilization.

VA transposition under microscopic visualization is approached cautiously, as manipulation of large, rigid, and frequently atherosclerotic arteries carries a substantial risk of brainstem infarction due to injury of critical perforators [3, 4]. Conventional microscopic MVD is technically challenging in VA-related conflicts because the microscope’s limited “tubular” field of view can hinder identification of perforators concealed behind the bulky vessel, rendering transposition hazardous and increasing reported complication rates to up to 25% [2]. In contrast, endoscopy improves visualization of short perforators and branch anatomy, enabling safer mobilization and potentially more durable long-term outcomes. In VBDE with VA-related conflict, simple interposition may be insufficient because of its caliber and pulsatility. However, fully endoscopic VA transposition for TN has not yet been described in the literature. This article describes an endoscopic strategy for safe VA transposition through improved visualization of the REZ corridor and branch vessels.

## Description of the technique

### Positioning and set-up

**Fig. 1:**
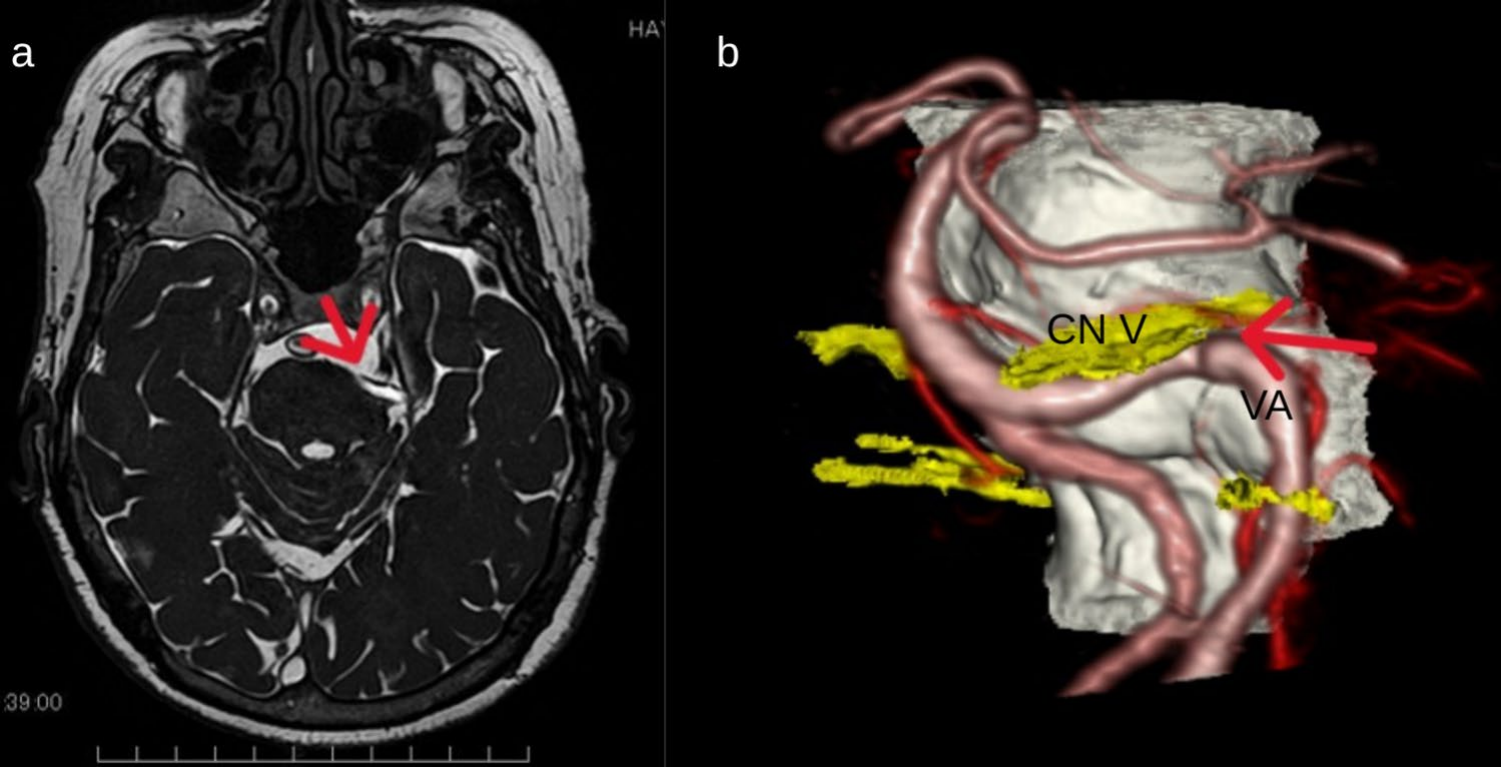
Preoperative neuroimaging of trigeminal neuralgia caused by vertebral artery compression. **a** MR cisternography demonstrating the neurovascular conflict (red arrow); **b** Three-dimensional fusion image showing the left vertebral artery adjacent to the root entry zone of the trigeminal nerve (red arrow). CN V, trigeminal nerve; VA, vertebral artery

After park-bench positioning, brainstem auditory evoked potential (BAEP) monitoring is initiated. Preoperative multimodal MRI image fusion maps the vertebral artery loop relative to the trigeminal nerve and helps anticipate variations that may narrow the standard corridor (Fig. 1a, b). A 4-mm 0° rigid endoscope attached to a holding arm maintains a panoramic view, freeing both hands for microsurgical maneuvers.

### Practical step-by-step surgical technique

**Fig. 2:**
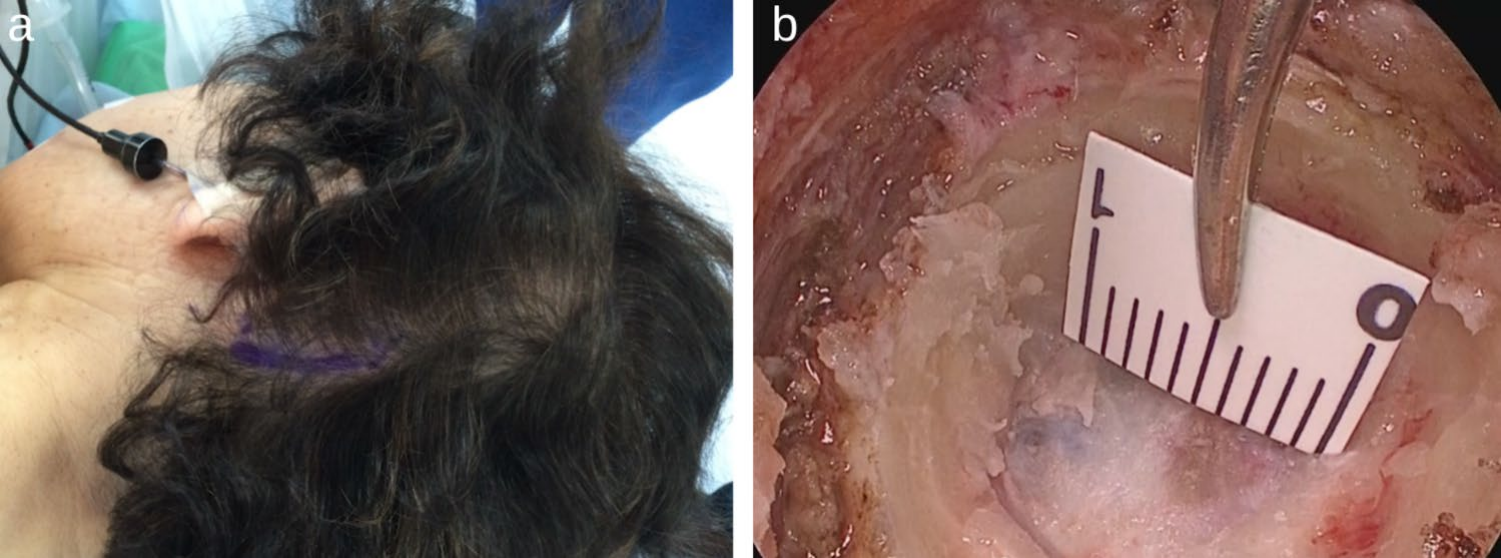
Operative set-up and keyhole retrosigmoid exposure. **a** Skin incision and operative positioning with brainstem auditory evoked potential (BAEP) monitoring. **b** Keyhole retrosigmoid craniotomy with a burr-hole diameter of approximately 1.5 cm

Endoscopy enables a minimally invasive approach using a 3.5-cm skin incision and a 15-mm craniotomy (Fig. 2a, b).

**Fig. 3:**
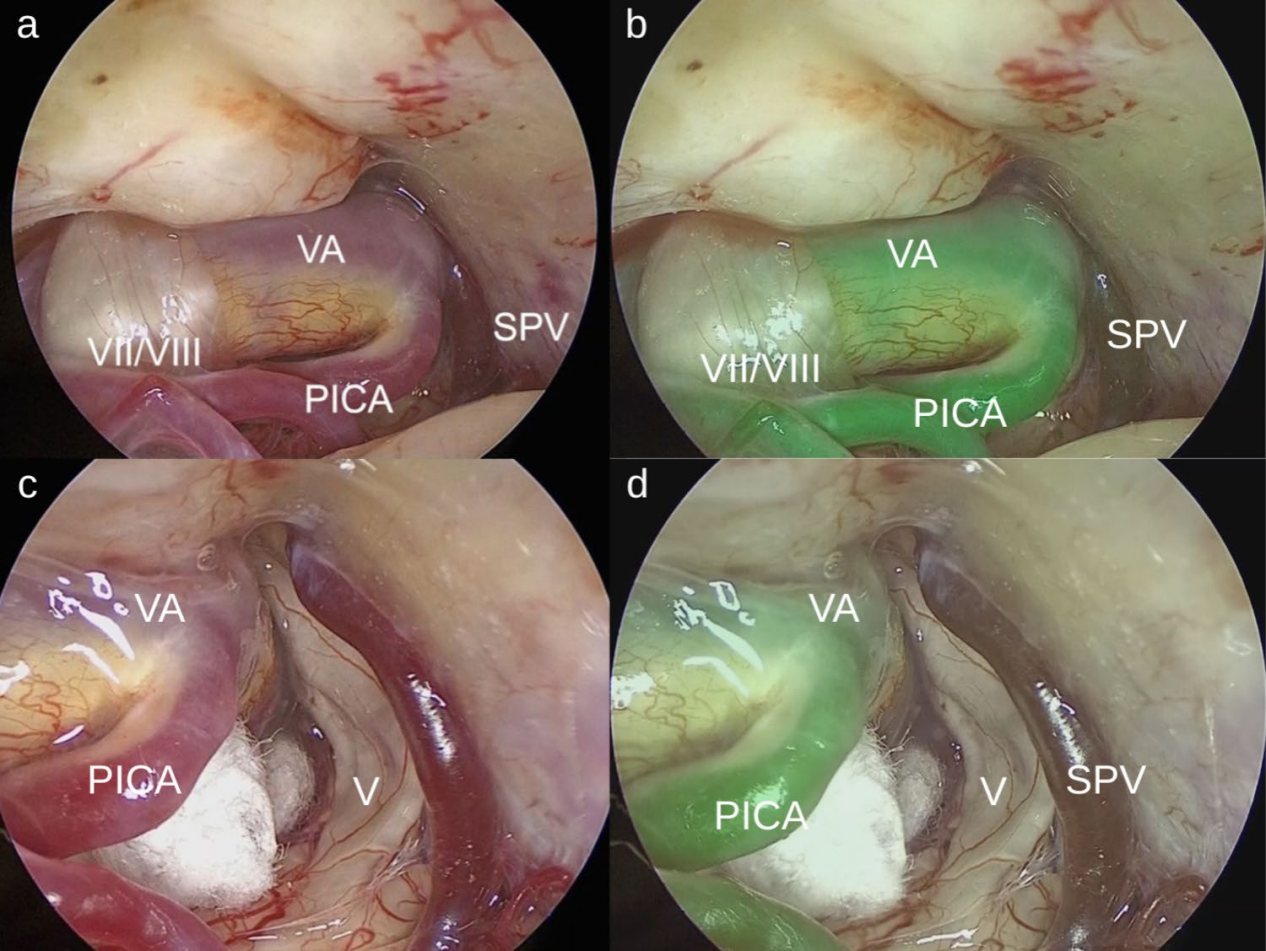
Key intraoperative steps of eMVD for TN due to the VA. **a** The VII/VIII nerve complex is displaced by a tortuous VA. **b** After ICG injection, the left VA and the origin of the left PICA were visualized, with identification of the SPV. **c** VA was secured to the petrosal dura mater with fibrin glue, additional shredded Teflon was placed between VA and the brainstem. **d** Post-decompression ICG angiography confirmed the absence of neurovascular conflict at the REZ. V, trigeminal nerve; VII/VIII, acousticofacial bundle; VA, vertebral artery; SPV, superior petrosal vein; PICA, posterior inferior cerebellar artery

After sealing the exposed bone with bone wax, the dura is opened with a U-shaped incision based on the sinus. With the endoscopic approach, fixed cerebellar retraction is avoided. The cistern is opened by sharply incising the outer arachnoid membrane, followed by gradual CSF release to achieve cerebellar relaxation. After identifying the vestibulocochlear nerve (CN VIII) and the superior petrosal vein, the endoscope is advanced through the corridor between them toward the trigeminal nerve. In this case, CN VIII was displaced posteriorly by a tortuous VA, and the standard endoscopic trajectory was occupied by a loop of the left VA (Fig. 3a, b). After gentle mobilization of the VA, the CN V was visualized at the REZ, where it was compressed, consistent with Grade III according to Sindou’s classification [5]. The endoscopic viewing angle allowed confirmation that no VA perforators were present within the intended transposition corridor and facilitated assessment of the anterior spinal artery.

Following ICG injection, the left VA and the origin of the left posterior inferior cerebellar artery (PICA) were visualized, confirming the absence of short perforators and the anterior spinal artery (ASA) within the intended transposition corridor (Fig. 3b).

Transposition was performed, and the VA was secured to the petrosal dura mater with fibrin glue between Meckel’s cave and the internal auditory canal. Given the limited space available for vertebral artery (VA) fixation and the caliber of the vessel, additional shredded Teflon was placed to reinforce the transposition (Fig. 3c). During this step, the absence of perforators within the manipulation field was confirmed. Neurovascular contact was successfully eliminated, and no indirect nerve compression was observed. Post-decompression ICG angiography confirmed the absence of neurovascular conflict at the REZ, excluded compression by an additional vessel, and verified patency of all branches of the mobilized artery (Fig. 3d). Meticulous inspection of the operative field, hemostasis, and irrigation of the surgical cavity are essential. The dura is closed in a watertight fashion, cranioplasty is performed using bone paste, and the soft tissues and skin are closed in layers.

### Indications

Fully endoscopic vertebral artery transposition is indicated for TN caused by VA compression at the CN V REZ, particularly when complete separation of the offending vessel from the nerve is required.

### Limitations

Endoscope-only VA transposition in VBDE-related TN is limited by the local branch anatomy and VA mobility. Short perforator tethering or an ASA in the intended corridor may preclude safe transposition and mandate interposition.

### How to avoid complications

Safe VA transposition requires circumferential assessment of the ectatic artery and the intended transposition corridor before mobilization. Panoramic endoscopic visualization and ICG angiography should be used to identify the origin of major branch vessels and to confirm the absence of short perforators within the working corridor. Fixed cerebellar retraction should be avoided; gradual CSF release provides adequate working space while minimizing traction on CN VIII. The VA should be mobilized only to the extent necessary to achieve complete decompression of the REZ. Final endoscopic inspection, supplemented by ICG angiography, should confirm elimination of neurovascular contact, absence of indirect compression, and preservation of arterial branch patency.

### Specific information for the patient

The aim of surgery is to relieve trigeminal neuralgia by separating the vertebral artery from the trigeminal nerve at its root entry zone. In VBDE-related cases, the feasibility of transposition depends on the anatomy of the perforators and the anterior spinal artery. If safe transposition is not possible, interposition may be performed instead.

## 10 key points summary

1. Preoperative preparation with multimodal image fusion supports trajectory planning, defines anatomy, and helps anticipate corridor distortion.
2. BAEP monitoring supports intraoperative safety during endoscopic manipulation in the CPA.
3. The procedure is initiated in the park-bench position through a minimally invasive keyhole retrosigmoid approach using a 3.5-cm skin incision and a 15-mm craniotomy.
4. Fixed cerebellar retraction is avoided by cisternal opening and controlled CSF release, creating a safer endoscopic working space.
5. Endoscopic panoramic visualization improves inspection of perforators and branch anatomy around the ectatic VA, which may be less reliably exposed through the more limited microscopic line of sight.
6. Pre- and post-decompression ICG angiography guide and validate the procedure by defining branch anatomy and demonstrating effective decompression with preserved patency of the transposed arterial branches.
7. VA transposition is the critical decompressive step in VBDE-related TN, with endoscopic visualization enabling complete separation of the offending vessel from the REZ.
8. Stable fixation of the mobilized VA is achieved using fibrin glue with Teflon reinforcement.
9. Careful hemostasis, irrigation, and watertight closure are key postoperative safety steps.
10. In complex VA-related conflicts, endoscope-only VA transposition requires a dedicated learning curve in endoscopic orientation.

## Supplementary Information

Below is the link to the electronic supplementary material.

ESM 1 Online Resource 1. Operative video demonstrating panoramic endoscopic microvascular decompression with VA transposition in VBDE-related TN. (MP4 62.8 MB)

## Data Availability

No datasets were generated or analysed during the current study.
